# Prescription antibiotics for outpatients in Bangladesh: a cross-sectional health survey conducted in three cities

**DOI:** 10.1186/1476-0711-13-15

**Published:** 2014-04-22

**Authors:** Mohitosh Biswas, Debendra Nath Roy, Afsana Tajmim, Sheikh Shahriar Rajib, Mosharraf Hossain, Fahamida Farzana, Nelufar Yasmen

**Affiliations:** 1 Department of Pharmacy, University of Rajshahi, Rajshahi 6205, Bangladesh; 2 Department of Pharmacy, Jessore University of Science and Technology, Jessore 7408, Bangladesh

**Keywords:** Antibiotics, Prescription patterns, Antibiotic resistance, Bangladesh

## Abstract

**Background:**

Antibiotics prescribing by physicians have gained due importance across the globe, mainly because of an increase in antibiotic usage, prevalence of infections and drug resistances. The present study is aimed to evaluate the physicians prescribing pattern of antibiotics, their usages by outpatients and disease conditions for which the antibiotics are prescribed in three cities of Bangladesh.

**Methods:**

This cross sectional health survey was carried out with a self designed standard questionnaire by manual data collection over a three months period (20.03.2013 to 20.06.2013) at three adjacent cities Jessore Sadar, Monirampur and Keshabpur upazila respectively. The data were collected from the patient’s prescription and by directly interviewing the patients who were prescribed at least one antibiotic during the study period. WHO Anatomical Therapeutic Chemical (ATC) classifications for antibiotics was used and descriptive statistics were applied to the collected data and analyzed using Microsoft Excel software. Modified Wald method was applied to calculate 95% CI.

**Results:**

A total of 900 prescriptions were analyzed during the study period. It was found that the prescriber prescribed antibiotics to the patients who were suffering mainly from cold and fever, infections, diarrhea and gonorrhea. The highest prescribed antibiotic groups were cephalosporins (31.78%), macrolides (27.33%), quinolones (16.33%), penicillins (7.11%), and metronidazoles (6.78%) respectively. Two or more antibiotics were prescribed in 25.44% of prescriptions. A total of 66.89% prescriptions had complete information on dosage form, 57% had complete direction for antibiotics use and 64.22% patients completed full course of antibiotics. Although 83% prescriptions have no clinical test for using antibiotics, even though the percentages of patients’ disease recovery were 61.78% and incompliance were 38.22%.

**Conclusion:**

From this research, it is observed that physicians prescribed antibiotics rationally in some cases but needs to ensure in all cases of prescription. Because irrational use leads to the spread of bacterial resistance to antibiotics and related health problems, our findings have important implications for public education and the enforcement of regulations regarding the prescription of antibiotics in Bangladesh.

## Background

The remarkable discovery of penicillin by Sir Alexander Fleming in 1928 was the beginning of antibiotic revolution, which changed the course of modern medicine [1]. Antibiotics have effectively prolonged the life expectancy and are currently the most commonly prescribed drugs in hospitals, worldwide [2]. But, excessive and inappropriate use of antibiotics renders increased drug resistance [3-5]. The rational use of antibiotics is a major health need.

In a developing country like Bangladesh, the cost of health care is a key cause for concern. The practitioners should be made aware of the importance of combination therapy in the treatment of certain infections so that the chance of resistance development can be ameliorated to the most possible extent. Many studies have implicated that the antibiotics are among the major group of drugs, which cause adverse drug reactions (ADRs) [6]. Bangladesh has made substantial progress in drug manufacturing since the promulgation of ‘Drug Control Ordinance-1982’ but irrational use, inappropriate prescribing and unjustified self medication of antibiotics often increase the cost of therapy and the risk of emergence of resistant organisms. Many doctors in Bangladesh are prescribing antibiotics irrationally without taking consideration the clinical test in most cases. Subsequently the patients are not completing the complete dosage regimen of antibiotics, if it is given in cold and general fever or even in other complicated infectious diseases.

The study of prescribing pattern of antibiotics infers to monitor, evaluate, and suggest modifications in the practitioner's prescription habits so as to make patient care reasonable and effective [7]. The knowledge about antibiotic utilization patterns is necessary for a constructive approach to problems that arise from multiple antibiotic usages. It is extremely important that institutions and hospitals should have an antibiotic policy and ensure that the best choices are made by individual prescribers [8]. A highly representative data aid the prescribers in rational antibiotic use and can improve the quality of patient care. This further envisages the need for the current study.

The aim of this survey based research is to analyze and justify whether antibiotics are being prescribed rationally or irrationally for outpatients as well as to indicate the prevalence of most prescribed antibiotics in three adjacent cities Jessore Sadar, Manirampur and Keshabpur of Jessore district in Bangladesh.

## Methods

### Study design, setting and study population

The present research is a cross-sectional prospective study carried out in three adjacent cities named Jessore Sadar, Manirampur and Keshabpur upazila in the district of Jessore under Khulna Division of Bangladesh. The data were collected for over three month’s period from March, 2013 to June, 2013.

Jessore is located in the southwestern tip of Bangladesh. Jessore Sadar is the main city of Jessore district located at 23.1681°N 89.2042°E and has a total area of 435.22 km^2^. It has a population of 742,898 where males constitute 52.85% of the population, and females 47.15%. This Upazila's eighteen-up population is 281,108 and has an average literacy rate of 44.2% (7+ years) [9]. Manirampur upazila is an upazila of Jessore District in the Division of Khulna, Bangladesh. It is located at 23.0167°N 89.2333°E and 16 km distant from Jessore city. Manirampur is the Second largest upazila of Bangladesh with an area of 444.72 square kilometres (171.7 sq mi). It is bounded by Jessore Sadar upazila on the north, Kalaroa and Jhikargachha upazilas on the west, Abhaynagar upazila on the east, and Dumuria and Keshabpur upazilas on the south. The total population of Manirampur is 326,093. Males constitute 51% of the population and females 49%. The density of population is 733/km^2^. The total people of eighteen years or older (18 or 18+) is 168,903 and has an average literacy rate of 29.1% (7+ years) [10]. Keshabpur is located at 22.9042°N 89.5667°E and has a total area of 258.53 km^2^. The distance from Jessore City is 32 km. Keshabpur has a population of 200,229. Males constitute 51.16% of the population, and females 48.84%. This upazila's eighteen up population is 103,794 and has an average literacy rate of 55.5% (7+ years) [11].

In this health survey any patient aged ‘0’ years to over 60 years whom prescribed one or more antibiotics at any stage during this study period is defined as an ‘antibiotic patient’. The term ‘antibiotic’ is used for ‘anti-infective for systemic use’ (antibacterials-J01 and anti-mycobacterials-J04), as classified by World Health Organization Collaborating Center (WHOCC) for Drug Statistics Methodology [12].WHO Anatomical Therapeutic Chemical (ATC) classifications for antibiotics [13,14] is used in this study.

### Data collection

This cross-sectional health survey was carried out with a self designed standard questionnaire by directly interviewing the 900 outpatients, 300 from each city respectively. Five students of the Department of Pharmacy and one faculty of Department of Pharmacy of Jessore University of Science and Technology were assigned and given instruction by the principle investigator, Mohitosh Biswas, Lecturer of Pharmacy Department of Rajshahi University for conducting this health survey. Written consent was taken from each patient during this study. Data were collected from the patients by random selecting the patients who came to buy the drugs from the pharmacies. The data collectors were waiting in front of the pharmacy shop and convince them to produce their prescription data to the interviewers as well as participated in the interview session. The patients who were unconscious/mentally retarded, who were suffering with psychiatric diseases and who were admitted into hospitals were excluded from the study. Few questionnaires were excluded during the data analysis because of inadequate information.

### Ethical considerations

The study was conducted following the general principles (section 12) of WMA declaration of Helsinki. This survey based research is also logistically supported by the Department of Pharmacy, University of Rajshahi. The human subjects involved in this study did not use any hazardous agents and samples were not collected from them. As the human subjects only participated in the interview, this survey based research didn’t take any further approval from institutional ethics committee.

### Statistical analysis

Descriptive statistics were applied to the collected data using Microsoft Excel software. Results are expressed graphically in percentages, mean, standard deviation (SD) and 95% CI. Modified Wald method was applied to calculate 95% CI.

## Results

**Figure 1:**
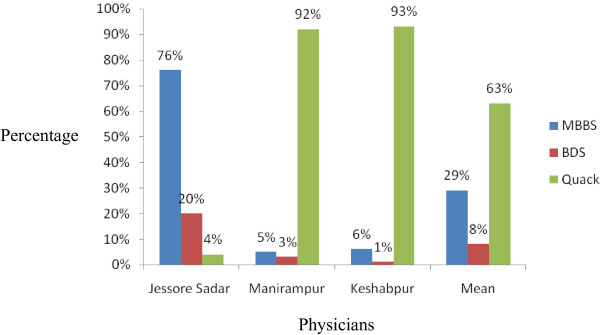
Prescriptions obtained from different health care professionals.

**Figure 2:**
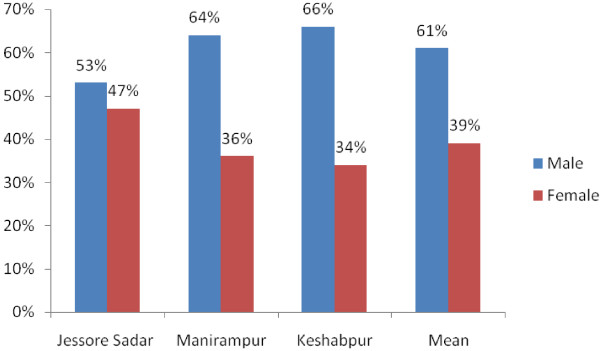
Gender variability of collected prescriptions.

**Figure 3:**
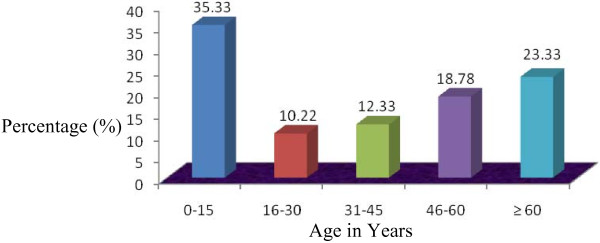
Age distribution of collected prescriptions.

**Figure 4:**
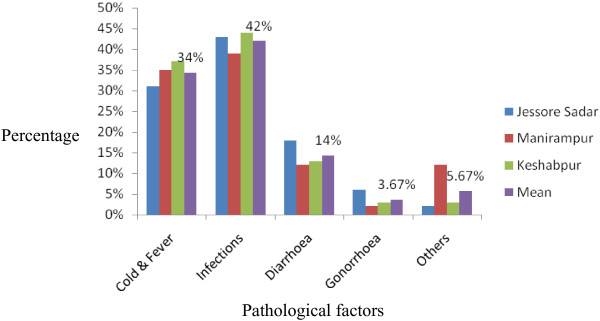
Reasons for visiting doctors.

This is the first survey in the Jessore Sadar, Manirampur and Keshabpur upazila indicating the prescription antibiotics. From this health survey study it is found that in these “three cities” averagely 29% patients visited Bachelor of Medicine, Bachelor of Surgery (MBBS) doctors, 63% visited Quack doctors whereas only 8% visited Bachelor of Dental service (BDS) doctors shown in Figure 1. There are variations in the results obtained from the cities. In Jessore Sadar, the highest percentage of patients (76%) visited MBBS doctors but in Manirampur and Keshabpur highest percentage of patients visited Quack doctors which were 92% and 93% respectively. The lowest percentage of patients paid a visit to BDS doctors in all the cities, Figure 1. Among the patients, 61% were male and 39% were female. In this study, males were prescribed 22% more antibiotics than females, Figure 2. The highest percentage of males (66%) was found in Keshabpur whereas the highest percentage of females was found in Jessore Sadar, Figure 2. Children aged from ‘0’ years to 15 years old took the highest percentage of antibiotics (35.33%) followed by older peoples aged 60 years or over 60 years (23.33%) whereas young people took the least percentage (10.22%) of antibiotics, Figure 3. The reasons for taking the antibiotics were due to suffering from infections (42%), cold and fever (34%), diarrhea (14%), gonorrhea (3.67%) and others diseases (5.67%), Figure 4.

The average highest prescribed antibiotic groups in these three cities were cephalosporins (31.78%, 95% CI: 31.56-32), macrolides (27.33%, 95% CI: 25.75-28.90), quinolones (16.33%, 95% CI: 15.78-17.34), penicillins (7.11%, 95% CI: 6.71-7.51), and metronidazoles (6.78%, 95% CI: 6.56-6.99) respectively, Table 1. Cephalosporins were prescribed highest in Keshabpur upazila (34.33%) whereas lowest in Manirampur upazila (29.67%).This antibiotic was prescribed for the patients suffering mainly from infections like respiratory tract infections (RTI), urinary tract infections (UTI), cesarean patients, typhoid fever etc. After the cephalosporins, the highest antibiotic usage was macrolides which were prescribed highest in Keshabpur upazila (41%) whereas lowest in Jessore Sadar (8.33%) and prescribed for curing cold and fever. Quinolones were prescribed highest in Jessore city (28.33%) and lowest in Keshabpur (6%) and prescribed for the ailment of typhoid fever, respiratory infections and gonorrhea. Penicillins were prescribed highest in Jessore city (12%) but lowest at Keshabpur (3.67%) and given mainly for recovery of wound infections as well as respiratory infections. On the other hand, Metronidazoles were prescribed highest in Manirampur upazila (9%) followed by lowest in Jessore city (4.33%) and prescribed for curing diarrhea and dysentery, Table 1.

**Table 1:** Frequency of prescribed antibiotics

	**TNP ( N = 900)**			**IP (%)**			**AVP (%)**	**SD**	**95% CI**
**Group of antibiotics with ATC code**	**JES (n = 300)**	**MAPR (n = 300)**	**KEPR (n = 300)**	**JES**	**MAPR**	**KEPR**			
Cephalosporins (J01D)	94	89	103	31.33	29.67	34.33	31.78	1.93	31.56 to 32
Quinolones (J01M)	85	44	18	28.33	14.67	6.00	16.33	9.19	15.28 to 17.34
Macrolides (J01F)	25	98	123	8.33	32.67	41.00	27.33	13.86	25.75 to 28.90
Aminoglycosides (J01G)	5	3	4	1.67	1.00	1.33	1.33	0.27	1.29 to 1.36
Penicillins (J01C)	36	17	11	12.00	5.67	3.67	7.11	3.55	6.71 to 7.51
Antituberculers (J04A)	20	2	1	6.67	0.67	0.33	2.56	2.91	2.23 to 2.89
Tetracyclins (J01A)	6	4	8	2.00	1.33	2.67	2.00	0.54	1.94 to 2.06
Metronidazoles (J01XD01)	13	27	21	4.33	9.00	7.00	6.78	1.91	6.56 to 6.99
Antifungals	6	11	8	2.00	3.67	2.67	2.78	0.68	2.70 to 2.86
Other antibiotics (J01X)	10	5	3	3.33	1.67	1.00	2.00	0.98	1.89 to 2.11

Here, JES=Jessore Sadar, MAPR=Manirampur, KEPR=Keshabpur, TNP=Total Number of Prescriptions, IP=Individual Percentage, AVP=Average Percentage, SD=Standard Deviation calculated by MS Office Excel-2007, 95% CI= Confidence Interval calculated by modified Wald method at 95% Confidence level.

Single antibiotic was prescribed in 74.56% of prescription whereas two or more antibiotics were prescribed in 25.44% of prescription. A total of 66.89% prescriptions have complete information on dosage form, 57% has complete direction for antibiotics use and 83% prescriptions have no clinical test for prescribing antibiotics. A total of 64.22% patients completed full course of antibiotics and the percentage of disease recovery was 61.78% whereas significant percentage of patients (38.22%) complained side effects after taking the prescribed antibiotics, Table 2.

**Table 2:** Prescription and usages pattern of antibiotics in three cities of Jessore district in Bangladesh

**Question Pattern**	**Response pattern**	**Frequency**	**Percentage (%)**
		**n = 900**	
Pattern of antibiotics	Single antibiotic	671	74.56
Prescription	Multiple antibiotics	229	25.44
Information on dosage form	Complete	602	66.89
	Incomplete	234	26
	Not mentioned	64	7.11
Information about the direction for antibiotic use	Complete direction	513	57
	Incomplete direction	387	43
Clinical test for prescribing antibiotics	With test	153	17
	Without test	747	83
Completion of full antibiotic course	Yes	578	64.22
	No	322	35.78
Patient’s compliance	Disease recovery	556	61.78
	Incompliance	334	38.22

## Discussion

Highest percentage of patients (76%) visited MBBS doctors in Jessore city because of the presence of Jessore Sadar Hospital where found MBBS easily and being the main city of this district, a lot of MBBS doctors practice here. On the other hand, Manirampur and Keshabpur is upazila of Jessore district which is 16 and 32 kilometers away from the main city respectively. Due to low facilities and life status, very few MBBS doctors are found here that is reflected in the survey results. Quack doctors are abundant here and patients have to pay minor fees for their visit, as a consequence highest percentage of patients, 92% and 93% visited Quack doctors in the Manirampur and Keshabpur upazila respectively.

Males were prescribed 22% more antibiotics than females. Higher prevalence of antibiotics in males also had been observed in previous studies conducted in Nepal and Bangladesh [7,15]. These findings can’t be fully explained although we think medical decisions are being biased by the male because of their dominance character in the Bangladesh. Antibiotics prescription rates in this study was found to be particularly high in the pediatric and geriatric populations perhaps because these populations are more prone to infections [16]. Guidelines suggest receiving antibiotic use only if positive infection was observed [17] but 83% prescriptions in this survey has no clinical test for positive microbial test although the physicians are prescribing antibiotics irrationally. Furthermore, established guidelines suggest that antibiotics should not be the choice of treatment in most diarrhea cases [18].

In our research it was found that cephalosporins accounted 31.78% of total antibiotic prescriptions which is high as compared to the study conducted in Nepal and Turkey but low than India [7,8,19]. The highest uses were by cefixime, cefuroxime and ceftriaxone for respiratory infections and other infections. This probably explains why ceftriaxone and cefixime have abnormally high resistances [20,21]. Acute respiratory infection was the condition associated most frequently with prescription antibiotic use, a result which substantiates findings from other Asian countries [8,22,23]. Our results are also consistent with findings in China, where low-severity illness was a major reason for giving children antibiotics [22]. This is probably a result of aggressive marketing policies of Bangladeshi Pharmaceutical Company on the physicians combined with inadequate knowledge of current treatment guidelines. The highest prescribed quinolones were levofloxacin, sparfloxacin and ciprofloxacin which is high compared to study conducted in India and Nepal [7,8].

In Bangladesh many doctors are not prescribing antibiotics by following the prescription guidelines of antibiotics. As a result, sometimes antibiotics are prescribed irrationally here to give quick relief of the patients without taking consideration of the patient’s disease condition. This is because antibiotics are the most commonly used and misused drugs by patients and prescribers [24]. Although the physician prescribe maximum antibiotics (83%) for outpatients in Bangladesh without clinical test and without giving complete direction for antibiotic use (43%), even though the percentage of patients disease recovery (61.78%) is satisfactory. This is because of the physicians long years service experiences as well as the broad spectrum nature of the prescribed antibiotics. This kind of antibiotic prescription habit of physicians may increase the misuse of antibiotics and resistance as well. Hospitals also account for antibiotic misuse worldwide due to non evidence based practice [25-29]. Our survey based research also reveals that significant percentage of patients receiving antibiotics in Bangladesh which is relevant to the reports on antibiotic usages in other parts of Asia, Europe or America [15,16,30,31] because antibiotics are considered as the second most prescribed drugs in the world, only next to the drugs indicated for cardiovascular diseases [32]. In a study undertaken in Vietnam in 1997, researchers discovered that more than 70% of patients were prescribed with inadequate amounts of antimicrobials for serious infections. In Turkey, 15-20% of all prescribed drugs are antibiotics. In China, researchers found that 63% of antimicrobials selected to treat proven bacterial infections were simply the wrong choice. The same is true even for the countries like Canada and the United States which developed their antibiotic usage control mechanisms. In these countries, it is estimated that physicians over-prescribe antibiotics by 50% [19].

This study has some limitations. The findings obtained from this small sample size (900 only) cannot be generalized to the whole population of Bangladesh. To better study this issue, future research should focus on both urban and rural areas and should involve the patients as much as possible. Additionally, seasonal variations in illnesses should also be taken into consideration, because they may have affected disease patterns and antibiotic use. Despite these limitations, our findings have significance in concern of current prescription antibiotics and their usage patterns in Bangladesh.

## Conclusion

Because irrational use of antibiotics leads to the spread of bacterial resistance to antibiotics and related health problems, our findings have important implications for public education and the enforcement of regulations regarding the prescription of antibiotics in Bangladesh. The study also urges the physician to be more professional and careful when antibiotic is prescribed for the outpatients. Effective strategies should be taken by the Government of Bangladesh to reduce the use of antibiotics which could include the development of policies to support the judicious use of antibiotics, strengthen the control of antibiotics selling and implement educational campaigns for prescribers.

## Competing interests

The authors declare that they have no competing interests.

## Authors’ contributions

MB was responsible for verbal training of the data collectors, supervision of data collection, data analysis, and has drafted the manuscript. DNR co-supervised the data collection from the cities whereas SSR, Md. MH, FF, NY and AT collected data from the survey cities. DNR contributed to the data analysis and statistical analysis. All authors have read the manuscript to revise it carefully and have approved the final manuscript.

## Authors’ information

**Mohitosh Biswas;** Lecturer, Department of Pharmacy, University of Rajshahi, Rajshahi-6205, Bangladesh.

**Debendra Nath Roy;** Assistant Professor, Department of Pharmacy, Jessore University of Science and Technology, Jessore-7408, Bangladesh.

**Sheikh Shahriar Rajib, Md. Mosharraf Hossain, Fahamida Farzana, Nelufar Yasmen and Afsana Tajmim;** Student, Department of Pharmacy, Jessore University of Science and Technology, Jessore-7408, Bangladesh.
